# How Shall We Best Impart Dental Instruction to Patients?

**Published:** 1870-09

**Authors:** George A. Mills


### HOW SHALL WE BEST IMPART DENTAL INSTRUCTION TO PATIENTS?

BY GEORGE A. MILLS.

**Read Before the Second District Dental Society, Brooklyn, N. Y., January 11th, 1870.**

This subject presents itself to us in the form of a sharp question, and each one who attempts to reply will do it in accordance with his present state of development in theory and practice. I hope that each one present will feel the importance of giving his convictions, or his experience and observation. If we will do this, I believe we will be better prepared to impart proper dental instruction to our patients.

I am asked to answer the question of my own proposition. Sixteen years of practice and watching have not been without their teaching. I was not inspired at the first entering our profession with that spirit, without which it is utterly impossible to make a sure and free advance, the spirit of love; which so tenaciously, and yet so kindly, absorbs *all* the difficulties which we are called to encounter. I do not say I am so fully imbued with this spirit of love, on which the very existence of this planet, "on which we live and move, and have our being," depends. It creates and gives form, until now, according to the design of the grand architect of Heaven, and do what we will, as badly as we may, and distort the beauties dispensed by His power, in spite of us, at the expense of our being put through the crucible, subjected to the intensest heat; for in His own time and in His own way, He will bring to pass that which He designed from the beginning.

We have accepted the calling of Dentists, some say dental surgeons, dental physicians, and oral surgeons. We all, more or less, assume to take the whole series of titles, and no one knows better than ourselves, how imperfectly fitted we are for the position. For I think we can not cheat ourselves. We are fully aware of what we know. Then to answer the question before us it is necessary, first, to fully understand what our patients need to know.

The first thing I can conceive that they need to know is the value of a Dentist who is honestly struggling to give his best services and time; or in other words, to show a better practice. But when it is said that the general practice is no better than twenty-five years ago then I must object, for facts show it to be far otherwise.

I think it safe to say that at no time in the history of our race was there ever bestowed so much of what is called dental service, as at the present day; and furthermore I do not hesitate to say that was three-fourths of this so called service not performed more teeth would be saved than are; and I am fearless to assert that never was there so much *faithful effort* in dental practice as now. And let me add here, I know faithfulness far transcends all other forms of advertising; although, unfortunately as it may seem to many, good growth is rather slow for this fast age. And yet every one of much experience will agree with me that it is far better to get good root before large branches appear; but the besetting sin of our profession is to put branches first.

Now there are principles on which we can all safely stand, and commence to impart dental instruction that will bring forth fruit, and that which is good. To instruct them properly we must be acquainted with what we desire to impart. I have been told by those of our profession that I must assume to know, and in that way I would carry such an assurance that would gain the confidence of those entrusted to my hands. Gentlemen, a greater heresy could not be taught. There is no power so great as the *plain truth*. It will always lodge conviction in the minds of those that seek the light. If our patients are to be taught the value of their teeth it must be done by the statement of such facts as will prove to them their importance and value. The mission of the dental profession is to teach them by precept and practice how their teeth can be retained, and act a large part in the prevention of many of the ills that arise from their loss. And here we need to be able to give an intelligent reason, so imparted that it shall be clear.

The days of mysteries are beginning to pass away, and the people are crying out for the facts, clothed in simple language; and what we need to know can be known by searching diligently. Another very important duty lies at the door of our profession: the teaching of parents their duty to the little ones committed to their charge, in the early care of the teeth. Thus preventing so many of the severe ills that arise from neglect. This teaching can result in the accomplishment of much good; then let us cry out against the great evil of the day, so wicked and unjust in this day of better lights, early extraction. In my opinion this can not be done in a more effectual way than by teaching face to face, and eye to eye, or in a conversational way.

If the proper and moral training of the children is the hope and safety of the nation, because of their easy direction in the right way, may they not by our proper teachings become powerful weapons of defense against the ruthless ravages of the early decay of teeth?

They should be very early taught the care of the teeth, and also furnished with such food as will of necessity require a vigorous mastication; which will do much towards a more perfect organization of the locality that embraces our specialty. We should impress on the minds of patients the strictest observance of cleanliness; which if done, as it might be, would do more towards prevention of decay than all the remedies of the present day in our possession. The necessity of the early care of the teeth by the Dentist, that he may assist in the prevention of the first stages of decay by the file, chisel, polishing materials, etc., correcting little irregularities which often, by the neglect of a few weeks or months, will result in serious hindrances to a healthy and proper development of these organs. This is best seen by practice. Our mission is (that we may be consistent) to *practice what we preach.*

And now let me say in closing, that the privilege we have of meeting in this organized association any others that it is the pleasure of some of us to be a part of is the open door of opportunity for the advancement of ourselves, by the instruction which is scattered broadcast among us. The warm sunshine of brotherly feeling that stands out so prominent in this society betokens a fruitful future. Let this day be one of improvement, and all others that may be ours, and the harvest will be rich in its rewards.

